# Multiple paths to health: A scoping review of dietary interventions for adults with Crohn’s disease and ulcerative colitis

**DOI:** 10.1177/17562848261479366

**Published:** 2026-08-13

**Authors:** Josephine Ingridsdotter, Vedrana Vejzovic, Eva Elmerstig, Klas Sjöberg

**Affiliations:** 1 Department of Care Science, Faculty of Health and Society, 621668 Malmö University, Malmö, Sweden; 2 Centre for Sexology and Sexuality Studies, Faculty of Health and Society, 621680 Malmö University, Malmö, Sweden; 3 Department of Social Work, Faculty of Health and Society, 621667 Malmö University, Malmö, Sweden; 4 Department of Clinical Sciences, 5193 Lund University, Malmö, Sweden; 5 Department of Gastroenterology, 59565 Skåne University Hospital, Malmö, Sweden

**Keywords:** adults, Crohn’s disease, diet, inflammatory bowel disease, scoping review, ulcerative colitis

## Abstract

**Background:**

Patient and clinical interest in dietary management of inflammatory bowel disease (IBD) is growing, yet a comprehensive overview mapping how dietary interventions are defined, composed, and evaluated across Crohn’s disease (CD) and ulcerative colitis (UC) remains scarce.

**Objectives:**

This scoping review aims to map current evidence on dietary interventions that may have an impact on the disease course in adults with CD or UC, thereby providing guidance for the design of forthcoming dietary interventions.

**Eligibility criteria:**

Included are English articles published between 2000-2025 investigating dietary treatments for adults (+18) reporting on symptoms, remission, or disease activity. Excluded are paediatric studies, single food/supplement-only interventions, and studies combining new medications with diet.

**Sources of evidence:**

Systematic searches of PubMed, CINAHL, Scopus, and Web of Science, originally conducted in January 2025 and updated in May 2026, identified 49 eligible studies.

**Charting methods:**

Data were charted using Covidence with a form guided by the TIDieR checklist. Dietary composition across interventions was visualised using heatmap analysis.

**Results:**

A range of dietary approaches were identified. Most improved patient-reported outcomes and disease activity indices, though a disconnect often persisted between symptomatic relief and inflammatory biomarker normalization. Despite variability even among nominally identical diets, heatmap analysis of diet composition revealed strong consensus around elimination of processed foods and added sugars, alongside emphasis on whole foods, fruits, vegetables, and unsaturated fats.

**Conclusions:**

Evidence points toward several possible diets for IBD, and clearly toward the Mediterranean diet and shared whole-food principles as a well-supported, safe, and guideline-endorsed foundation. Next step is designing rigorous research with standardised outcomes, disease-specific populations, mechanistic sub-studies, and the implementation support that adherence requires.

## Plain language summary

**Why was the study done?:**

Both patients and healthcare professionals are interested in how to manage inflammatory bowel disease (IBD) using diet. However, it is still unsure of what these different diets look like and how well they work.

**What did the researchers do?:**

Research published between 2000 and 2025 was reviewed to map out the different dietary approaches tested in adults with IBD. The researchers looked at 49 studies to see how the diets were defined, what foods were included and excluded, and what effects they had on symptoms and inflammation.

**What did the researchers find?:**

While many diets helped improve how the patients felt, this improvement did not always match up with a reduction in gut inflammation measured by lab tests. Importantly, despite the variety of diet names, most of them shared common principles; cutting out processed foods, added sugar and sugary drinks, and instead a focus on eating whole foods such as fruits, vegetables containing fibre, and healthy fats found in nuts and olive oil.

**What do the findings mean?:**

The evidence points to several diets that may be helpful for managing IBD. However, the Mediterranean diet stands out because it is well-supported by research and considered safe. The next step is to conduct more rigorous research that uses standard methods to measure results and better understand how diet works to help manage IBD. For now, focusing on a varied diet containing whole foods appears to be a beneficial strategy.

## Introduction

Inflammatory bowel disease (IBD), including Crohn’s disease (CD) and ulcerative colitis (UC) is a chronic inflammatory condition associated with significant distress and negatively impact quality of life (QoL). During disease relapses, patients frequently experience physical symptoms, heightened anxiety and depression, and disruptions to social functioning.^1^ While certain QoL domains may improve during remission, evidence suggests that residual effects persist, resulting in lower QoL relative to the general population.^2,3^

IBD may develop at any age, with incidence rates peaking between 15 and 24 years and again between 55 and 64 years for both CD and UC.^4^ Historically, the diseases have been most prevalent in Europe and North America; however, incidence is rising in regions previously considered low-risk, including Africa and Asia.^5–7^ The etiology of IBD remains unclear, but current evidence indicates that genetic susceptibility, combined with environmental factors such as diet or pollution, can trigger abnormal immune responses to gut bacteria or food, resulting in chronic intestinal inflammation.^5,8^ Traditional treatments primarily focused on symptom suppression, whereas contemporary approaches aim to induce and maintain remission with mucosal healing to prevent complications.^9^ Biologics have revolutionised IBD treatment, but challenges persist. Some patients do not respond at all, and others suffer a loss of response, where patients initially respond well but later relapse.^10^ In parallel with pharmacological advances, major professional bodies such as the European Crohn’s and Colitis Organisation (ECCO) and the American Gastroenterological Association (AGA) increasingly recognise food and nutrition as an important adjunct option in IBD.^11,12^

Dietary therapy for IBD is a relatively recent development, with exclusive enteral nutrition (EEN) representing a good example with rather positive outcomes, particularly for CD. EEN has demonstrated efficacy in inducing remission among paediatric patients, achieving outcomes comparable to corticosteroids but without their associated adverse effects.^13^ EEN contributes to inflammation control and beneficially modulates the gut microbiota, thereby promoting mucosal healing.^14^ However, similar efficacy has not been observed in adult CD or UC populations.^15^ Due to the unpredictable course of IBD and persistent uncertainty regarding dietary roles, many patients independently alter their eating patterns based on personal experience rather than clinical guidance, resulting in confusion and inconsistent dietary practices.^16–18^ Self-imposed dietary restrictions in IBD have also been associated with increased risk of malnutrition.^19^

In response to this growing clinical and patient-driven interest, research on dietary interventions such as the Specific Carbohydrate Diet (SCD), Crohn’s Disease Exclusion Diet (CDED), and the Mediterranean diet (MD) has grown. To date, the best clinical recommendations derive from the AGA Clinical Practice Update^12^ and the ECCO consensus guidelines,^11^ both of which emphasise the potential of diet while also acknowledging gaps in evidence related to diet definitions, composition, outcome measures, and disease-specific effects. While recent systematic and narrative reviews have reported the clinical efficacy of these various dietary interventions^20–26^ there is still a lack of a comprehensive overview that also explores how specific diets are defined, dietary composition (permitted and prohibited foods) in similarly named diets, and how outcomes are measured across studies. Different reviews have reported heterogenous results, with variation in methodologies, and target populations. Even interventions bearing the same name may differ substantially in composition and application, further complicating the interpretation of results.^25^

Furthermore, much of the existing literature treats IBD as a single entity, despite increasing evidence that certain dietary interventions may benefit CD but not UC, and vice versa.^25,27^ A comprehensive scoping review mapping these variations across both conditions may clarify areas of concordance and divergence, thereby supporting more nuanced clinical application consistent with ECCO and AGA priorities.

Therefore, this scoping review aims to map current evidence on dietary interventions that may have an impact on the disease course in adults with CD or UC, thereby providing guidance for the design of forthcoming dietary interventions.

## Method

This scoping review followed the framework of Arksey and O’Malley^28^ with refinements by Levac et al.^29^ and was reported according to Preferred Reporting Items for Systematic reviews and Meta-Analyses extension for Scoping Reviews (PRISMA-ScR) guidelines (Supplemental material 1). A scoping review was selected for its suitability for exploratory objectives and possibility to accommodate diverse study designs. The process followed five stages: (1) identifying the research question; (2) systematically searching the literature; (3) selecting eligible studies; (4) extracting and charting the data; and (5) collating, summarising, and reporting the findings. A protocol has been submitted.

### Identifying the research question

Together with the research team and in consultation with a research librarian, the following research question was identified: ‘What dietary interventions have been studied in adults with IBD, CD, or UC to maintain or induce remission?’ A PICOS framework was developed to provide structure, Table 1. The question was considered to strike a sufficient balance between specificity and breadth, encompassing both named diets and broader dietary approaches relevant to IBD management.

**Table 1.** Eligibility criteria for study selection, structured according to the PICOS framework (Population, Intervention, Comparison, Outcome, Study characteristics).

PICOS tool	Determinants
Population	Late adolescents and adults (18+ years old) diagnosed with IBD, CD or UC:
Intervention	Dietary interventions (e.g., named diets or food-based therapies) aimed at managing IBD
Comparison	Any comparator
Outcome	Clinical outcomes related to symptom alleviation, remission rates and disease activity indices.
Study Characteristics	All study designs that investigate dietary interventions in adults with IBD (e.g., randomized controlled trials, cohort studies, case series).

### Identifying relevant studies

A systematic search was performed in January of 2025 in the databases PubMed, CINAHL, Scopus and Web of Science. Additionally, searches for grey literature using Google and Crohn’s and Colitis foundation were included. Reference lists were checked for additional citations until saturation was met. An updated search was conducted in May of 2026, using identical search strings to capture studies published in the intervening period.

The search strategy was developed iteratively in consultation with a research librarian, using keywords and synonyms combined with Boolean operators (see Supplemental Material 2). Keywords were related to three search blocks: inflammatory bowel disease (e.g., ‘inflammatory bowel disease,’ ‘Crohn’s disease,’ ‘ulcerative colitis’), dietary intervention (e.g., ‘diet,’ ‘dietary intervention,’ ‘dietary therapy’), and treatment outcomes (e.g., ‘remission,’ ‘disease activity,’ ‘inflammation’). An additional search block on age was included to reduce irrelevant results. Terms such as ‘adolescence’ and ‘young people’ were retained to capture publications on late adolescence (18–24 years) where the term ‘adult’ was not used explicitly. The full search strings for each database are provided in Supplemental Material 2.

### Study selection

Studies were eligible if they examined dietary interventions as treatment for IBD (CD or UC) in adults (18+), and if they reported on symptom alleviation, remission, or disease activity indices, and were published in English between 2000 and 2025. The year 2000 was selected as the start date as a preliminary scoping search revealed a marked acceleration in dietary IBD research from this point onward. Studies were excluded if they focused on children, animals, single food items, supplements, or if they were letters or editorials. Additionally, they were also excluded if they included mixed paediatric-adult populations from which adult-specific conclusions could not be distinguished. Finally, they were also excluded if they presented outcomes unrelated to IBD disease activity such as low Fermentable Oligo-, Di-, Monosaccharides And Polyols (FODMAP) outcomes limited to irritable bowel syndrome symptoms or if medication was introduced concurrently with the dietary intervention in a way that precluded attribution of effects.

**Figure 1.**
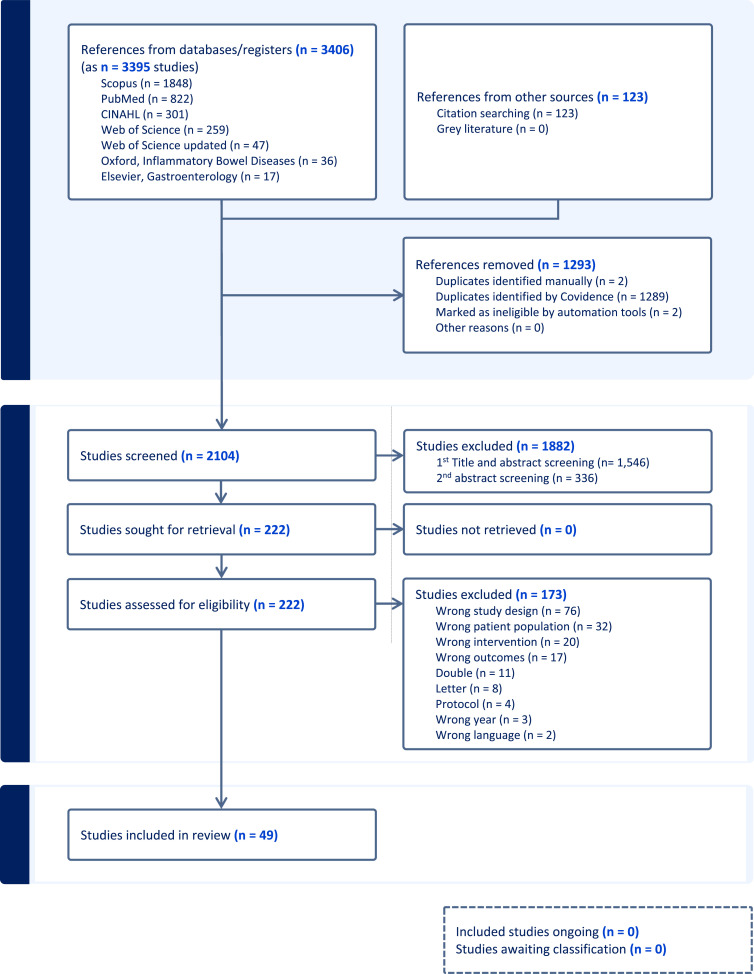
Prisma flow chart.

Titles were initially screened by the first author (JI) in Zotero to remove clearly irrelevant articles, before the remaining references were exported to Covidence for abstract and full-text screening. All authors participated at each screening stage, with each article independently assessed by two authors and conflicts resolved through consensus meetings involving an author who had not assessed that specific article (Figure 1). A total of 49 studies were included: 36 primary intervention studies, 8 reviews, and 5 case studies.

### Charting the data

Data was extracted using Covidence^30^ extraction tool guided by the TIDieR^31^ checklist. The charting form was developed by JI, piloted on a subset of included articles and refined iteratively, consistent with Levac et al.^29^ Charted data included general study characteristics (authors, year, country, design, aim), details of dietary interventions (type of data, delivery mode, duration, comparator, concomitant medication use, diet composition), and outcomes of interest. During the pilot testing, adherence and barriers or facilitators were included in the charting but was removed due to being out of the scope. For reviews: scope, study types included, key findings and identified research gaps were also charted. Two authors (JI and KS) conducted the pilot and JI completed most of the subsequent charting with regular consensus meetings with KS.

### Collating, summarising and reporting the results

Evidence was collated by grouping studies according to their primary dietary intervention. Where studies described a named, recognisable dietary protocol (e.g., CDED), this name was retained while studies describing broader dietary patterns (e.g., semi-vegetarian, plant-based) were grouped under the corresponding overarching approach (e.g., plant-based diets). Some overlap between groupings is therefore inherent. Study designs were categorised into randomised controlled trials (RCT), non-randomised and/or uncontrolled interventions, reviews, or case studies. To facilitate understanding of dietary composition across interventions, a heatmap was developed in R Studio using the pheatmap package, without scaling or hierarchical clustering. Food groups were coded as: (0) not mentioned; (1) explicitly included or recommended; (2) included with conditions or limitations; or (3) explicitly excluded. The conditional category captured nuances such as phased reintroduction protocols or individualised exclusions based on IgG antibody reactivity. Primary studies, case studies, and reviews are reported in separate tables (Tables 2–6).

**Table 2.** Characteristics and outcomes of included studies evaluating dietary approaches for induction of remission in active disease.

Author, year (country)	Study design	Population (n (m/f); medication status)	CD	UC	Comparator diet	Duration of intervention	Outcome: Measurement	Results
MD								
Lewis et al., 2021 (USA)	RCT	92 (29/63); concomitant medication	X	​	SCD	12 weeks	Induction of symptomatic remission: FCP, CRP, sCDAI, CDAI, sIBDQ	At week 6: Comparative remission rates did not differ, SCD (46.5%) and MD (43.5%, NS). Comparative FCP and CRP (NS). Within groups week 0 => 6, improvement in sSCDAI, CDAI, short IBDQ, fatigue, sleep interference, pain and social isolation (P < 0.02).
ErolDoğan et al., 2024 Turkey	RCT	48 (25/21); concomitant medication	​	X	MD, MD + curcumin, and MD + resveratrol	8 weeks	disease activity + QoL: Truelove-Witts index, CRP, ESR	Within-group comparisons showed MD, MD + curcumin, MD + resveratrol interventions reduced disease activity and inflammation and improved QoL (p < 0.05). Between-group comparisons was NS except pain subparameter of SF-36 and the MEDAS score (p < 0.05).
IgG								
Gunasekeera et al. 2016, United Kingdom	RCT	76 (32/44); concomitant medication	X	​	Sham diet	4 weeks	QoL + disease activity: SIBDQ, CDAI, HBI, FCP	Improvement in SIBDQ (P < 0.05), CDAI (P = 0.009) and HBI (P=0.01). FCP (NS).
Rajendran et al. 2011, United Kingdom	Non-Randomized and/or uncontrolled Intervention	29 (11/18); concomitant medication	X	​	N/A	4 weeks	Disease activity: mCDAI, ESR, CRP, albumin, IgG4	26 (90%) reported symptomatic improvement (P = 0.0001). ESR decreased 23=>17 mm ⁄ h (P = 0.021). IgG4 titres for excluded foods decreased a mean of 3015–2306 mcgA ⁄ l (P = 0.003). CRP and albumin changes (NS).
CDED								
Yanai et al. 2022, Israel	RCT	40 (18/22); concomitant medication	X	​	CDED + PEN, CDED alone	24 weeks	clinical remission + disease activity: HBI, CRP, albumin, weight, FCP, SES-CD.	Median FCP decreased week 0 => 12 (P=0.0123). 30 patients had high CRP, this decreased week 0 => 6 and was sustained to week 24 (14.5 => 8.4 mg/L, P=0.0378; =>8 mg/L P=0.0378). Albumin changes (NS). 22 patients had paired colonscopies at week 0 and 24, median SES-CD score 72.8% decrease (P=0.0025).
Szczubełek et al. 2021, Poland	Non-Randomized and/or uncontrolled Intervention	32 (14/18); concomitant medication	X	​	N/A	12 weeks	Inducing remission: CDAI, IBDQ, CRP, ESR	Clinical remission was 76.7% at 6 weeks and 82.1% at 12 weeks. CDAI and IBDQ dropped after 6 and 12 weeks (p < 0.001). CRP reduced (p = 0.021). ESR changes (NS).
Pasta et al. 2025, Italy	RCT	45 (30/15); concomitant medication	X	​	Traditional MD	24 weeks	symptomatic remission: HBI, FCP, CRP, ESR, fibrinogen, body composition, IBD-Q	Differences observed between groups in FCP, CRP, ESR and fibrinogen (NS). CDED had lower HBI (p = .027) and higher symptomatic remission rates (p < .0001) than controls.
Trakman et al. 2025, Australia	RCT	15 (10/5); concomitant medication	X	​	Sulfur reduction diet and a whole food diet.	12 months	Change in disease activity: CDAI, CRP, FCP, IBDQ-9	QoL improved (p < 0.001). CRP, FCP, and CDAI were reduced (p < 0.045)
AID								
Marsh et al. 2024, Australia	RCT	58 (27/31); concomitant medication	X	X	8-week ‘Eat for Health’ education followed by 8-week IBD-MAID education. => general healthy eating education	6 weeks (16 for comparator group)	QoL + disease activity + symptoms: SCCAI, CDAI, SIBDQ, PRO2, FCP, CRP	Week 0–8: No between-group differences (NS). Within-group, Intervention improved PRO2 (p=0.001), SIBDQ (p=0.004), FCP (p=0.002), and CD disease activity (CDAI p=0.03); UC disease activity (SCCAI NS). Comparator improved SIBDQ (p=0.019). Greater reduction in food additives correlated with lower FCP (p=0.05), PRO2 (p=0.04), and higher SIBDQ (p=0.03).
Exclusion diet								
Bartel et al., 2008, Austria	RCT	14 (9/4); concomitant medication	X	​	Control diet (low-fat, low-fiber, high-carbohydrate)	6 weeks	Intestinal healing: Sonography, Endoscopy, MRI	After 6 weeks 3/4 patients in active group & 1/9 in control group improved in MRI or endoscopy evaluation (75% versus 11%, P 0.027 by Fisher exact test)
Narula et al. 2022, Canada	Non-Randomized and/or uncontrolled Intervention	17 (8/9); concomitant medication	X	​	N/A	17 weeks (52 weeks for follow-up)	Adherence + clinical remission + clinical response: MARS-5, HBI, SES-CD, FCP	Patients initially on EEN 2 weeks. 13 deemed responders => 12 weeks of exclusion diet. Satisfactory adherence was reported among 11/13 (84.6%) week 6 and 8/12 (67%) week 14. 5 patients (38.5%) attained CR at week 2 and maintained until week 14. Endoscopic response in 6 (46.2%) participants and ER was seen in 2 (15.4%).
Sulfur reduction diet								
Ye et al. 2025, Canada	RCT	22 (10/12); concomitant medication	​	X	Habitual diet	8 weeks	Change in disease activity: pMayo, FCP, butyric acid, valeric acid, lipopolysaccharide-binding protein,	RS group more than ≥50% reduction in FCP compared to controls (p < 0.05). Lipopolysaccharide-Binding Protein levels changed over time (p < 0.05). Control group pMayo scores decreased from baseline to week 8 (p < 0.05), while the RS group showed a trend (p = 0.06). RS group trended towards higher increases in butyric acid compared to controls (p = 0.06). RS group showed higher increases in valeric acid compared to controls (p < 0.05).
Day et al. 2022, Australia	Non-Randomized and/or uncontrolled Intervention	28 (13/15); concomitant medication	​	X	N/A	8 weeks	Remission, symptoms: Mayo score, pMayo score, endoscopy, NHI, biochemical inflammation markers, GSRS, IBD-Control-8 IBD-FRQoL-29.	Clinical response observed in 13/28, endoscopic improvement in 10/28, histology in 12/28. Serum CRP (NS) FCP decreased by 131 μg/g (P = 0.02). FRQoL improved (P = 0.001).
Trakman et al. 2025, Australia	Non-Randomized and/or uncontrolled Intervention	7 (3/4); concomitant medication	​	X	CDED and a whole food diet	12 months	Disease activity: pMayo score, CRP, FCP, IBDQ-9	No inferential statistics were made due to small sample size. QoL were stable or increased (NS). CRP decreased (NS). Food additive score reduced more than fourfold (p = 0.013).
Fiber-modified diets								
Brotherton et al. 2014, USA	RCT	7 (1/6); concomitant medication	X	​	Control group excluded whole grains, dairy products, and spicy foods on symptomatic days, at least 48 ounces of fluid each day, but limit fluids to sips within 30 minutes of meals.	4 weeks	Symptoms + disease activity: IBDQ, HBI, CRP, ESR	Participants consuming whole wheat bran reported improved HRQoL (p = 0.028) and GI function (p = 0.008) CRP and ESR (NS).
AIP								
Chandrasekaran et al. 2019, USA	Non-Randomized and/or uncontrolled Intervention	15 (4/11); concomitant medication	X	X	Usual care switched to AIP, everyone their own control	6+5 weeks	Disease activity: SIBDQ, HBI, pMayo score, CRP, FCP	SIBDQ increased 46.5 => 61.5 (P= 0.03). CD disease activity (HBI), UC disease activity (pMayo), CRP, FCP improved too.
Konijeti et al. 2017, USA	Non-Randomized and/or uncontrolled Intervention	15 (4/11); concomitant medication	X	X	N/A	6 week elimination phase and 5 week maintenance phase (11 weeks)	Clinical remission: HBI, pMayo, CRP, endoscopy	UC disease activity (pMayo) improved week 0→6→11 (5.8→1.2→1.0, p=0.01). CD disease activity (HBI) improved week 0→6→11 (7→3.6→3.4, p<0.01). CRP (NS). FCP week 0→11 (471→112, p=0.12). Among 7 patients with week 11 endoscopy, improvements in SES-CD (n=1), Rutgeerts (n=1), Mayo endoscopy subscore (n=4).
Whole food diet								
Limketkai et al. 2025, USA	RCT	28 (15/13); concomitant medication	X	​	Habitual diet	​	Induction of remission: HBI, FCP, ESR, CRP, healthy eating index	The diet led to greater clinical remission (odds ratio [OR] 1.41; 95 % CI 1.03–1.93; P = 0.03) similar change in FCP. Adherence associated with clinical remission (OR 1.18; 95 % CI 1.02–1.38; P = 0.03) and FCP (−234; 95 % CI -465 to − 3; P = 0.047).
MD + LFD + PEN								
Narimani et al. 2024, Iran	RCT	46 (N/A); concomitant medication	​	X	control group, received dietary recommendations for healthy eating (no menu plans)	6 weeks	Diseae activity: IBDQ-9, SCCAIQ, IBQ-9, CRP, TAC and anthropometric variables	Disease activity decreased in combined diet group VS. controls (p= 0.043), and baseline (p< 0.001). QoL increased in the combined diet group VS. controls, and the baseline (p< 0.001). hs-CRP decreased in combined group (p< 0.01), increased in the controls (NS). TAC changes (NS).
Low/high emulsifier diet								
Fitzpatrick et al. 2025, Australia	RCT	24 (12/12); concomitant medication	X	​	Habitual diet	4 weeks	Induction of remission: HBI, GIUS, CRP, FCP, IBD-Q, VAS for symptoms, IBUS-SAS	Clinical remission (HBI <5) in 9/12 on HED and 7/12 on LED; IBUS-SAS 51 to 33 on HED (p=0.014) and 57>44 on LED (p=0.01). Bowel wall thickness reduced 34% on HED and 15% on LED. QOL & fatigue improved (p≤0.05).
Gluten-free diet								
Alborzi Avanaki et al. 2025, Iran	RCT	26 (7/19); concomitant medication	​	X	Placebo	6 weeks	Induction of remission: ESR, CRP, FCP, IBDQ, SCCAI	ESR, CRP and QoL improved but FCP increased (NS).

**Table 3.** Characteristics and outcomes of included studies evaluating dietary approaches for maintenance of remission.

Author, year (country)	Study design	Population (n (m/f); diagnosis; disease activity; medication status)	CD	UC	Comparator diet	Duration of intervention	Outcome: Measurement	Results
MD								
Lacerda et al., 2021 (Portugal)	RCT	52 (43/10); concomitant medication	X	X	Diet based on MD	4 weeks	Inflammation + symptoms: body composition, symptoms, CRP, FCP and zonulin.	For CD and UC combined, Reduction in weight, BMI, waist circumference and body fat between moment 1 and 3 (P = 0.05). Symptoms, CRP, FCP and ZO (NS).
Haskey et al. 2023, United Kingdom	RCT	28 (10/18); concomitant medication	​	X	Canadian Habitual Diet Pattern	12 weeks	Disease activity: SCCAI, FCP, CRP, pMayo score	Week 12, pMayo score [p = 0.008]. FCP for MD (NS). FCP increased for CHD from week 0 to 12 (P = 0.375).
PBD								
Arvidsson et al. 2024, Denmark	Non-Randomized and/or uncontrolled Intervention	15 (7/8); concomitant medication	X	​	N/A	12 weeks	Disease activity impact: HBI, IBDQ, IBD-F	HBI 3.1 to 2.4 from 0 to 12 w, p=0.028, IBDQ 173 to 191, p=0.006.
Limsrivilai et al. 2025, Thailand	Non-Randomized and/or uncontrolled Intervention	61 (39/22); concomitant medication	X	X	N/S	54 weeks	maintenance of remission: CDAI, Mayo score, SIBDQ, FCP	For CD and UC combined, Cumulative flare rate 22.6%. Increased fiber associated with lower flare rate (p=0.019). Significant difference between increased and decreas fiber intake groups (p=0.03). FCP (NS). Clostridia class increased in the fiber intake group.
Fiber-modified diets								
Fritsch et al. 2021, USA	RCT	18 (7/11); concomitant medication	​	X	Improved standard american diet with higher amounts of fruits, vegetables and fibre	10 weeks (2 wk washout period)	Maintaining remission: pMayo score, sIBDQ, and short-form 36 health survey, CRP, acetate, serum amyloid A.	Serum amyloid A (7.99 mg/L => 4.50 mg/L (P = 0.02), but NS compared to iSAD. FCP (3.23 mg/L => 2.51 mg/L, P = 0.07). Fecal levels of acetate increased (40 => 42 iSAD, 54 LFHFD (baseline vs LFHFD, P= 0.05; iSAD vs LFHFD, P =0.09).
AID								
Keshteli et al. 2022, Canada	RCT	53 (34/19); concomitant medication	​	X	Canada’s food guide diet	6 months	QoL + disease activity: SIBDQ, blood, urine, and stool samples (FCP, metabolomic analysis)	Clinical relapse occurred in 5 AID patients (19.2%) and 8 controls (29.6%). The subclinical response (FCP < 150 ug/g) higher in AID (p = 0.02). FCP trended lower for AID (p = 0.053, borderline NS) but increase for controls (p = 0.002). SIBDQ baseline to last visit for both groups (NS).
Preda et al. 2024, Romania	Non-Randomized and/or uncontrolled Intervention	168 (80/88); concomitant medication	X	X	Habitual diet	12 months	Remission maintenance: CDAI, Mayo score, FCP	Clinical remission maintained in 95.2% vs 85.7% (p=0.036); CD subgroup 95% vs 80% (p=0.044); UC NS. FCP higher at follow-up in controls (NS).
Exclusion diet								
Nitescu et al. 2023, Romania	Non-Randomized and/or uncontrolled Intervention	139 (79+60); concomitant medication	X	X	Habitual diet	6 months	maintenance of remission + compliance + disease activity: CDAI, Mayo score, CRP, endoscopy, CT enterography, ESR	Clinical remission at six months was maintained in all patients on the exclusion diet (100%). In the control group, 4 relapsed (1 UC, 3 CD) and 90 remained in remission (p=0.157). UC subgroup: 100% vs 97.4% (NS); CD subgroup: 100% vs 94.3% (NS). Baseline ESR higher in diet group: 29 vs 16 (p=0.019), similar at six months (p=0.440).
Low emulsifier diet								
Sandall et al. 2020, UK	Non-Randomized and/or uncontrolled Intervention	20 (6/14); concomitant medication	X	​	Habitual diet (their own controls)	14 days	Symptoms and disease activity: PRO-2 score, IBD-C-8, FR-QoL-29at, Education method usability scale	Frequency of consuming emulsifiers decreased by 94.6%. QoL improved (p=0.028), Sypmtoms reduced (p=0.006, and disease control scores improved (P=0.026).
MD + AID								
Marlow et al. 2013, New Zealand	Non-Randomized and/or uncontrolled Intervention	8 (2/6); no concomitant medication	X	​	N/A	6 weeks	Disease activity, microbiota composition: CRP, micronucluei numbers, gene and bacteria expression	CRP and micronucluei numbers (NS). 3.551 genes had altered expression, some directly relevant to inflammation (P < 0.05). Bacteria abundance trended toward normalisation (NS).
Low meat diet								
Albenberg et al. 2019, USA	RCT	213 (43/170); concomitant medication	X	​	High meat diet	49 weeks	Symptomatic relapse, higher dosage of medication or surgery required: sCDAI	High-meat participants consumed ≥2 servings of red or processed meat in 98.5% of weeks versus 18.8% in the low-meat group. Any or moderate-to-severe relapse occurred in 62% and 42% of participants, respectively, NS difference in time to relapse (any: P=.61; moderate/severe: P=.50)

**Table 4.** Characteristics and outcomes of included studies evaluating dietary approaches in mixed or dual-aim populations (induction and maintenance).

Author, year (country)	Study design	Population (n (m/f); diagnosis; disease activity; medication status)	CD	UC	Comparator diet	Duration of intervention	Outcome: Measurement	Results
IgG								
LiuJian et al. 2018, China	RCT	97 (50/47); concomitant medication	​	X	Sham diet	6 months	Symptoms: Mayo score, endoscopy	After intervention, pMayo lower in intervention group than in controls (P < 0.05). QoL improved after food exclusion (P < 0.05). Extraintestinal manifestations decreased: intervention 7=>2; control 6=>5. Endoscopy (NS).
Bentz et al. 2010, Germany	RCT	40 (16/24); concomitant medication	X	​	Sham diet	12 weeks (6 weeks each diet without washout)	Symptoms: Diary with questions (1-4 score) related to stool frequency, abdominal pain and general well-being	Daily stool frequency reduced by 11% compared to sham diet group (P= 0.004). Score (abdominal pain, general well-being and stool frequency NS).
PBD								
Chiba et al. 2010, Japan	Non-Randomized and/or uncontrolled Intervention	22 (14/8); concomitant medication	X	​	Omnivorous diet	43 to 82 days (median 49 d). Follow up after 2 years if induced remission	Induced and maintained remission: CRP, CDAI	16/16 in remission at 1 year and 15/16 in 2 years vs. 4/6 and 2/6, respectively (P=0.0003).
Lamers et al. 2022, The Netherlands	Non-Randomized and/or uncontrolled Intervention	26 (11/15); concomitant medication	X	X	N/A	6 months	Disease activity + symptoms: HBI, P-SCCAI, FCP	Clinical disease activity for CD (HBI) and UC (SCCAI), HRQoL, and FCP (NS). Improved diet quality was associated with lower impact of disease on daily life (P = .003) and less fatigue (P < .001) but clinical disease activity HRQoL, and FCP (NS).
Whole food diets								
Trakman et al. 2025, Australia	Non-Randomized and/or uncontrolled Intervention	42 (21/21); concomitant medication	X	X	CDED and a ulcerative colitis diet (sulfur reduction diet)	12 months	Maintenance of remission: CDAI, pMayo score, CRP, FCP IBDQ-9	QoL improved (p < 0.001). CRP, FCP, and (NS). CDAI and pMayo were reduced (p < 0.027).
Low FODMAP diet								
Bodini et al. 2019, Italy	RCT	55 (24/31); concomitant medication	X	X	Different amounts of FODMAP	6 weeks	Disease activity: HBI, Mayo score, CRP	CD disease activity (HBI) 4=> 3 (P = 0.024), UC disease activity (Mayo score) NS, FCP (77 => 50 P= 0.004)

**Table 5.** Characteristics and findings of case reports describing dietary approaches in individuals with inflammatory bowel disease.

Citation, year & country	Study design	Study population total (m/f), diagnosis + severity, medication	Duration of intervention	Outcome: Measurement	Results
AID					
Olendzki et al. 2014, USA	Case series	11 (4/7) IBD + active disease, concomitant medication	>4 weeks	Inducing remission and symptoms: HBI, MTLWSI	CD patients had a baseline HBI average of 11 => 1.5 after the diet. UC patients had a baseline MTLWSI of 7 (one missing) and a follow-up score of 0. Mean reductions were 9.5 (HBI) and 7 (MTLWSI).
PBD					
Sandefur et al. 2019, USA	Case study	1 male, CD + active disease, concomitant medication	>6 months	Remission: HBI, colonoscopy	No signs of CD on follow-up colonoscopy. HBI 0 indicating clinical remission with no medication.
Ketogenic diet					
Calabrese et al. 2025, USA	Case study	1 female, UC + active disease, concomitant medication	12 weeks	Induction of remission: pMayo score, Ulcerative Colitis Patent-reported outcome (UC-PRO)	Clinical remission after 3 weeks (PMS 0, UC-PRO 0).
SCD					
Khandalavala et al. 2015, USA	Case study	1 female, UC + active disease, concomitant medication	> 2 years	Remission, symptoms: endoscopy	Within 3–6 months, improvement in stools and abdominal pain. Within 6 months, daily life activities returned. Weakness and fatigue dissipated, weight remained stable. Colonoscopy 2 years => no inflammation. Biopsies confirmed remission.
Arjomand et al. 2022, USA	Case study	1 male, CD + active disease, concomitant medication	42 months	Remission: CRP, FCP, cytokine levels, lipid profile, and nutritional labs	From week 0-29, CRP (39.5 mg/L => 1 mg/L) from week 0-15, FCP (493 ug/g =>70 ug/g). Endoscopy showed ulcers, edema, and narrowing but at month 12 normal mucosa.

**Table 6.** Characteristics and findings of review articles addressing dietary approaches in inflammatory bowel disease.

Author citation, year (& country)	Condition(s)	Dietary intervention(s) investigated	Scope of evidence (no. Studies; years span)	Key findings/themes	Key limitations found	Research gaps highlighted
Systematic review						
Charlebois et al., 2016 (, Canada)	IBD	Low residue/low fiber diets and exclusion diets	12; 1985-2011	Exclusion diets and the low FODMAP diet show promise for having therapeutic benefits for patients with IBD.	NR	Further research using more precise methods of implementing the diets.
Comeche et al., 2020 (, Spain)	IBD	Low FODMAP diet, IgG diet, fiberrelated diets, PBD, microparticles diet, MD and MD + AID.	31; 1979-2020	CDAI improved (High heterogeneity). No differences were observed for ALB, FC, and CRP.	High heterogeneity	Further research is needed
Gleave et al., 2024 (Canada)	IBD	AIP, fiber-modified diet, 4-SURE diet, exclusion diets, SCD, MD, CDED, IgG4 diet.	10; 1995-2022	Diets share common elements, such as an initial elimination phase, subsequent reintroduction of dietary components, inclusion of whole foods, and exclusion of highly or ultraprocessed foods. Limited evidence to recommend a single diet.	High heteogeneity (Absence of control groups, standardized outcomes, variable adherence rates and tolerability, changes to medications, small sample sizes, and elements of bias.)	Larger, randomized studies with standardized methodologies and outcome measures, rigorous adherence assessment, and an emphasis on endoscopic assessment outcome measures are required to validate most diets that have been studied for IBD.
Jaramillo et al., 2023 (, USA)	UC	AID, PEN, low FODMAP, SCD and fixed diets	9; 2019-2022	Diet changes may prevent recurrence in UC patients in remission. Significant systemic changes in the microbiota with AID. CD patients tolerated MD and SCD. a FODMAP diet + counselling + regular follow-up could help persistent GI symptoms in quiscent IBD.	NR	Rigorous, larger, randomized controlled trials of nutritional therapies for maintaining remission in UC
Narrative review						
Boneh et al., 2023 (, Israel)	CD	CDED	14; 2014-2023	CDED can serve as an alternative to EEN in many adult cases, especially mild–moderate disease. For complicated cases, CDED should be considered case-by-case and more evidence is needed.	High heterogeneity (control diets, study design, follow-up duration, and outcome reporting)	(1) Identification of predictors of response (2) CDED as sole maintenance vs. combined therapy (3) Flexibility of CDED for different patients (4) Indications for EEN over CDED (5) Combining CDED with PEN (6) Which foods are likely to trigger flares? (7) Cost and geographic barriers for CDED (8) Strategies to improve dissemination and training
Istratescu et al., 2024 (Romania)	IBD	CDED, PBD, low-fat diet, low-meat diet, IgG diet, AID, Exclusion diet, PEN	NR; NR	No single diet to recommend for IBD remission maintenance. IBD patients self-administer diets, even those with lacking evidence. There is risk with restrictive diets, and several diets for IBD may promote unhealthy eating behaviors.	NR	NR
Clinical review						
Liu and Day 2024 (, New Zealand)	IBD	PBD	23; 2010-2022	Eleven case reports and eight single-arm trials. Semi-vegetarian diets most studied plant-based approach. Most reported PBD as safe and effective, but their role as adjunct or sole therapy remains uncertain.	Heterogeneity found (Few controlled data, small samples, inconsistent adherence reporting)	Further research needed. Rigorous interventional designs with proper controls and consistent, meaningful outcome reporting.
Scoping review						
Soublette Figuera et al. 2025, Canada	IBD	Studies examining the intake of UPF and/or food additives	1997-2025	Evidence suggests potential links between UPF and food additive intake and IBD outcomes.	Heterogeneity in UPF definitions, variability in additive formulations, and the lack of standardized dietary assessment methods limit definitive conclusions.	Integrate standardized exposure assessment methods with validated outcome measures to better clarify the role of UPF and food additives on IBD trajectory.

## Results

### General characteristics

Included for review were 49 studies comprised of RCTs and non-Randomised and/or uncontrolled Interventions (n= 36), reviews (n= 8) and case studies (n= 5) published between 2008 and 2025. Studies originated predominantly from North America (n=19), Europe (n=16) and other regions including Australia, Israel, Iran, New Zealand, Japan, China, Thailand and Turkey.

Regarding the dietary interventions, patient populations included those with active disease (aimed at inducing remission) and those in quiescent state (aimed at maintaining remission). Most studies specified that patients were on stable concomitant medication, meaning that the diet was used as an adjunct therapy. In some cases, the dietary intervention resulted in a lower dose.^32,33^ While many studies included patients with a history of treatment failure, some specifically focused on biologic-naïve populations.^34,35^ To ensure high adherence, several studies utilised professional catering or meal delivery for the initial 6 to 12 weeks of the trial.^36–39^ Some studies also provided key foods rather than the full meals.^40–43^ For instance, Marlow et al.^41^ provided participants with salmon, organic avocados, extra virgin olive oil, and green tea to supplement their diet. Many interventions relied on detailed educational materials with structured menu plans and recipe books.^44–48^ Other interventions often combined dietary recommendations with ongoing remote support through booklets, mobile apps, phone calls or social media groups.^34,49–54^ Detailed characteristics of individual studies including population, intervention protocol, comparator, outcomes and key findings are presented in Tables 2–6.

### Summary of dietary interventions

The identified literature encompasses a range of dietary approaches, from general recommendations for a certain type of diet to targeted exclusion protocols (see Tables 2–5). Studies are organised by clinical goal: Table 2 presents studies targeting induction of remission in active disease, Table 3 presents studies targeting maintenance of remission, and Table 4 presents studies enrolling mixed populations or addressing both aims.

The most frequently studied were The Mediterranean diet (MD), immunoglobulin antibody (IgG and IgG4) guided diets, and the Crohn’s Disease Exclusion Diet (CDED). The MD^37,42,55,56^ characterised by minimally processed whole foods such as vegetables, fruits, fish, olive oil and lower intakes of red meat and added sugar, while the IgG and IgG4 guided diets^35,52,54,57^ eliminate specific foods based on elevated levels of IgG antibodies to create tailored individual dietary plans. The underlying hypothesis is that these specific foods trigger a delayed immune response that contributes to the perpetuation of gut inflammation.^35^ The Crohn’s Disease Exclusion Diet (CDED)^34,53,58,59^ is a phased (elimination, reintroduction, maintenance) diet that besides this design also removes dietary components hypothesised to adversely affect the gut microbiome and immune system such as certain additives and processed foods, pro-inflammatory fats, refined carbohydrates and caffeine or alcohol. It is often accompanied with partial enteral nutrition (PEN). For both MD and IgG guided diets, in active CD the studies demonstrated an improvement in patient-reported outcomes, but the impact on inflammatory markers was less uniform. A similar pattern was seen in active UC for IgG and IgG4 diets with endoscopic assessment not showing improvement, while both patient-reported outcomes and inflammatory markers improved with the MD. CDED studies focused exclusively on active CD populations and assessments in endoscopy, histology in addition to disease activity indices showed improvement and mucosal healing. Notably, IgG and IgG4-guided approaches were studied exclusively in active or mixed disease populations, with no evidence specifically examining their role in maintenance of remission.

Anti-inflammatory diets (AID), exclusion diets, plant-based diets (PBD) and reduced sulfur (RS) diets were also frequently represented. AID^38,44,60^ typically recommends whole foods while restricting processed foods, refined carbohydrates, and certain proinflammatory fats. Similarly, the exclusion diets consisted of removing western diet components^48,61^ and restriction to intensively monitored organic foods^43^ while PBD^32,39,51,62,63^ emphasises plant foods with variable inclusion of fish or other animal products depending on the protocol. RS diets^46,58,64^ were designed to reduce dietary sources of sulfur, thereby limiting colonic hydrogen sulfide production by sulfate-reducing bacteria. Excess hydrogen sulfide is suggested to impair intestinal barrier integrity by breaking disulfide bonds in the gut mucus layer, a mechanism particularly implicated in UC pathophysiology. RS diets were examined exclusively in UC patients with active disease, with promising but preliminary results including clinical and endoscopic improvement, though one study^58^ was limited to descriptive analysis due to small sample size. Studies with AID included both CD and UC populations, examining both active disease and remission maintenance, and reported reduced relapse rates and improved QoL, though findings were largely derived from non-randomized or uncontrolled interventions. Exclusion diets were examined in both CD and UC, with some studies reporting maintenance of remission. PBD studies focused predominantly on CD but also included a mixed CD and UC maintenance study,^63^ with reports of sustained remission and reduced relapse rates. While another study^62^ examined PBD for induction of remission in active CD, controlled trial data were limited overall.

Furthermore, some studies focused on fibre-modified diets^36,40^ where fibre quantity, type is adjusted and/or refined carbohydrate and fat intake is limited to influence disease activity, while the Auto-Immune Protocol (AIP),^49,50^ is also a phased diet that removes dietary components suggested to trigger the immune system such as grains, eggs, nightshade vegetables, sugar, legumes and dairy. The specific carbohydrate diet (SCD)^37,65,66^ was also assessed which restricts complex and refined carbohydrates except monosaccharides. Whole food diets^58,67^ emphasise minimally processed nutrient-dense foods while restricting additives. Low emulsifier diets^68,69^ restrict foods containing emulsifiers and thickeners hypothesised to adversely affect the gut microbiome and barrier function. Fibre-modified, whole food diets and low emulsifier diets addressed both active disease and remission maintenance, while AIP and SCD (one RCT and two case studies) focused exclusively on active disease. Fibre-modified, whole food diets, AIP, and SCD approaches reported symptomatic improvements for both CD and UC, with mixed effects on inflammatory markers. Low emulsifier diets, both examined in CD, showed improved QoL and symptoms in a short-term feasibility study in quiescent disease, while an RCT in active CD found improvement in both low and high emulsifier groups with no clear advantage to restriction.

The remaining literature addressed hybrid and emerging interventions in both active disease and remission maintenance. One study assessed the clinical impact of low versus high meat intake^70^ finding no significant difference in relapse rates. A low FODMAP diet^47^ examined patients in remission and active disease, showing some effect for CD but not for UC. A hybrid intervention combining MD, PEN and low FODMAP principles^45^ reduced disease activity and improved QoL in UC patients with active disease, while a combination of MD and AID^41^ in CD patients in remission reported significant altered gene expression related to inflammation but no significant changes in inflammatory markers. Furthermore, a single case report described clinical remission in a patient with active UC following a ketogenic diet,^71^ with normalisation of symptoms after three weeks. Finally, a gluten-free diet^72^ in active UC showed trends towards improvement in some inflammatory markers and QoL, while FCP trended in the opposite direction. Two further emerging interventions, published after our predefined search cutoff, warrant mention as addenda. A randomised controlled trial of time-restricted feeding (TRF, an intermittent fasting approach confining food intake to a defined daily window) in adults with CD in remission with overweight/obesity (n = 35) found significant reductions in BMI, visceral adiposity, and clinical disease activity compared to controls, alongside favourable shifts in adipokines and gut microbiota composition.^73^ Additionally, a large randomised controlled trial (n = 97) of a fasting-mimicking diet (FMD, a plant-based, low-calorie, low-protein diet designed to mimic the physiological effects of fasting while still providing nutrients) in adults with active mild-to-moderate CD found significantly higher rates of clinical response and remission, alongside reductions in fecal calprotectin, compared with baseline diet.^74^

### Summary of existing reviews

Eight reviews were identified that investigated dietary interventions for IBD, either both conditions or CD and UC alone. The reviews included literature published between 1979 and 2023. While many of these studies explored different diets including MD, low FODMAP diet, IgG and IgG4 guided diets and AID, some were more specific in their scope focusing specifically on CDED, efficacy and safety of PBD and only one ultra-processed or additive restricting diets.^75^ The included reviews identified potential therapeutic benefits for the different diets but with high uncertainty. The reviews cited that limitations included small sample sizes, inconsistent reporting and a lack of standardized outcome measurements. Comeche et al.^25^ also highlighted that while there was symptomatic improvement, these changes were not reflected in objective biomarkers such as CRP or FCP. Furthermore, Istratescu et al.^76^ noted the potential risks with highly restrictive diets which may lead to unhealthy eating habits or nutritional deficiencies. To bridge the gaps, the authors of the reviews emphasize that larger, randomized trials that utilise precise methods of implementations and rigorous assessments are needed. Furthermore, to facilitate head-to-head comparisons, future research should standardize outcome measures. One further systematic review and meta-analysis of the MD in IBD, published after our predefined cutoff, is noted here as an addendum.^77^ Pooling eight studies in predominantly adults, it reported a pooled clinical remission rate of 62% with the MD, with comparable efficacy between CD and UC, but found no significant advantage over control diets overall. Consistent with the other reviews summarised above, the authors highlighted a high risk of bias across included studies and the absence of endoscopic or histological outcome data.

### Diet composition across interventions

**Figure 2.**
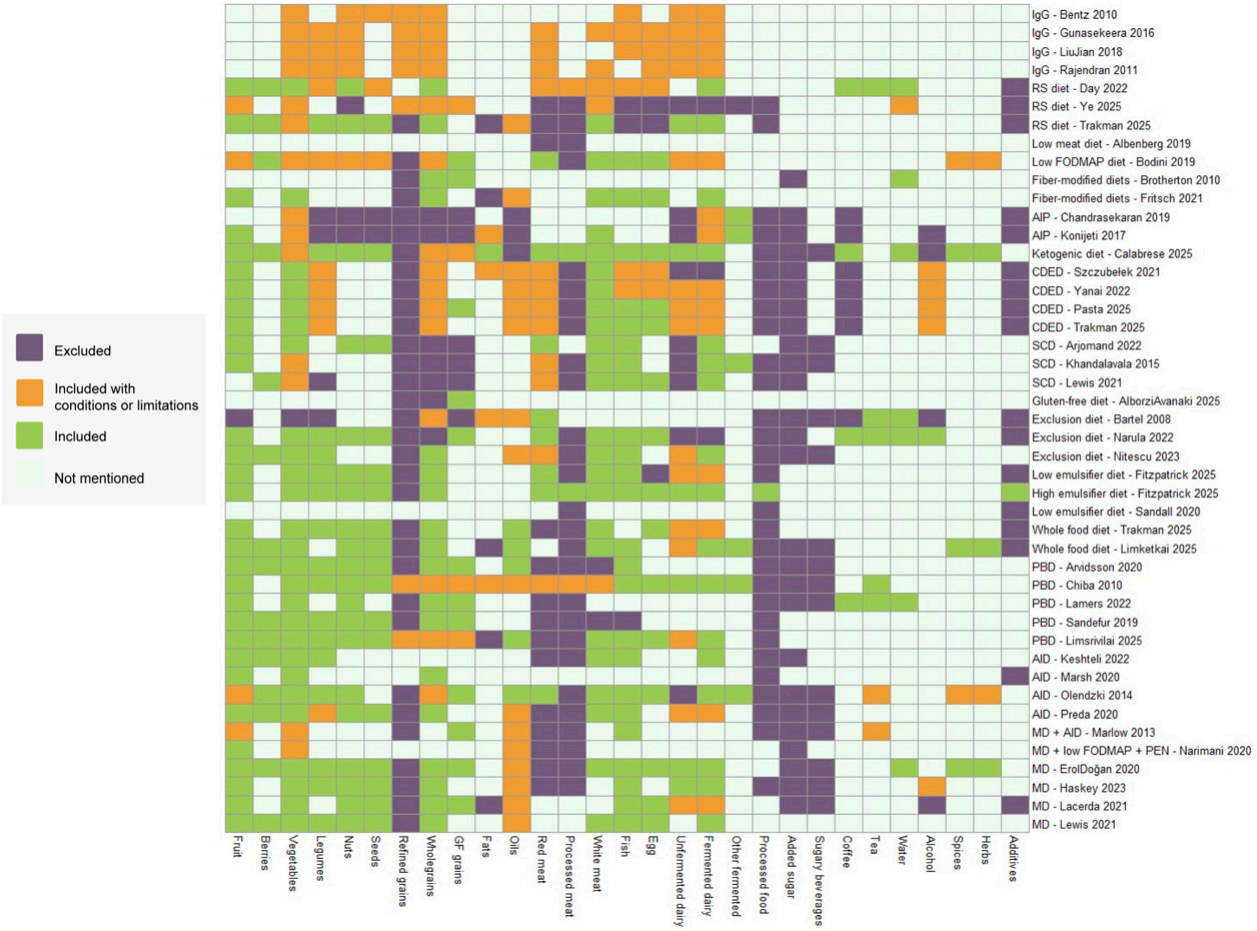
Heatmap of dietary composition by study and dietary approach, indicating whether each food component was excluded, included with conditions or limitations, included, or not mentioned in the dietary protocol.

The different recommendations are stated in Figure 2. As can be seen there are some discrepancies between the studies. Seven studies, one version of AID,^33^ AIP^49,50^ and CDED^34,53,58,59^ introduced foods through phases. These interventions typically began with a highly restrictive elimination phase with certain allowed foods and/or PEN/EEN to induce symptomatic relief, followed by a ‘maintenance phase’ where no foods were introduced, or a systematic ‘reintroduction phase’ where food groups were gradually added back based on individual tolerance. Similarly, one study introduced food after one week of fasting^62^ and another one after two weeks of EEN.^48^ In contrast, IgG and IgG4 guided diets made all food groups in these interventions classified as conditionally included in the heatmap, reflecting that inclusion or exclusion was determined on an individual basis.

Some interventions applied specific restrictions to food groups. For example, AIP excluded nightshade vegetables,^49,50^ two RS diets excluded cruciferous vegetables^58,64^ and two SCD interventions restricted starchy vegetables.^37,66^ Other diets, like the CDED^34^ and one version of the AID,^33^ employed a phased approach where the texture of fruits and vegetables was modified during a flare, with whole forms being gradually reintroduced as symptoms improve. One intervention^43^ excluded fruit and vegetables entirely due to only using food derived from intensively monitored organic farming, and organic production of fruits and vegetables was considered intrinsically difficult to control. Legumes varied by restrictions in amount^34^ type (Lentils or split peas in one SCD^37^) or preparation method (skinned or long term cooked^61^).

Regarding fats, many emphasised olive oil such as in the MD^37,45,56^ and whole food diets.^58,67^ Basis for excluding fats or oils was for saturated fats, or often due to omega-6 fatty acids specifically in “corn, safflower, sunflower oil, margarine” with Konijeti et al.^49^ and Haskey et al.^56^ noting these fatty acids are linked in disease progression.

Processed foods could include canned, industrially frozen, dried or anything ready-made with an expiration date.^37,43,48^ Several studies also focused on excluding food additives such as carrageenan,^38,42,46,48,64,68^ artificial sweeteners and maltodextrin,^38,68^ sulphite/sulfate,^46,48,58,64^ nitrite/nitrate,^46^ carboxymethyl cellulose,^42,68,69^ lecithin,^48,68,69^ or gum.^48,69^ While most studies excluded added sugars, several had honey as an exception that could be used to sweeten food.^33,41,48,61,65,66^ One of the RS diets also limited sulfate through drinking water, so identifying the water source was necessary.^64^

Several interventions included components beyond food recommendations. Two MD^42,55^ prescribed daily curcumin (2 g/day of curcumin and 1600 mg/day of curcumin respectively) while the latter also included 500 mg/day of resveratrol supplementation. To address nutritional adequacy. One version of the SCD was combined with iron, vitamin B12, vitamin D3 and a daily multivitamin,^65^ another study provided intramuscular multivitamin injections every three weeks^43^; and some diets were paired with PEN.^53^

## Discussion

This scoping review aimed to map current evidence on dietary interventions studied in adults with CD and UC that can alter the disease course and is based on 49 articles. The identified dietary approaches were diverse, with the majority appearing to improve patient-reported symptoms and QoL. Despite substantial heterogeneity, certain pattern emerged across the varying approaches: the elimination of processed, foods, added sugars and food additives alongside an emphasis on whole foods. A recurring finding was improvement in disease activity indices without a concurrent normalisation of inflammatory markers; a pattern whose interpretation forms a central focus of this discussion.

### Multiple paths to improved health

Most of the frequently studies interventions such as the MD, CDED, AID, PBD, RS diets, and AIP each appeared to offer some benefit, though through different proposed mechanisms and with different levels of supporting evidence. Despite surface-level differences, the heatmap analysis revealed substantial overlap. The consensus is less about what to eat than what to stop: processed foods, added sugars, sugar-sweetened beverages, and various food additives (particularly emulsifiers, artificial sweeteners, and preservatives). Instead, there is a lesser consensus what to include*:* minimally processed foods including fresh fruits and vegetables, unsaturated fats from sources such as nuts, seeds, and olive oil, and fermented dairy products. This convergence aligns with epidemiological and emerging evidence linking processed foods, added sugars, sugars-sweetened beverages and excess animal protein to IBD risk,^78–81^ and with the broader “food matrix” concept^82^ which recognises that health outcomes are shaped by complex interactions within whole dietary patterns rather than isolated nutrients. Importantly, these shared dietary features align closely with general healthy eating recommendations from national food agencies^83–85^ and the ECCO guidelines^11^ on dietary management of IBD.

Rather than requiring that patients adopt one optimal diet, the present study suggests that multiple dietary approaches, the MD, PBD, AID, and more targeted protocols like RS diets, CDED or AIP, may offer overlapping pathways to improved health, potentially through shared mechanisms despite their nominal differences. There may be multiple paths to Rome. The implication for clinical practice is that dietary guidance can be meaningful without prescribing a single diet.

### Disease specific patterns

A key objective of this review was to map evidence for dietary interventions separately for CD and UC, as the two conditions have different pathophysiology, and research has shown they can react differently to diet. However, some included studies^42,63^ enrolled mixed IBD populations and reported results as combined data without disease-specific subgroups, limiting disease-specific conclusions for those studies and reflecting a broader pattern of conflation in dietary intervention research.

Recent mechanistic evidence demonstrates that diet influences inflammation through distinct microbiome pathways in each condition: in CD, specific bacterial taxa and metabolites mediate dietary effects, whereas in UC microbial richness and global community composition are the primary drivers of protection.^86^ CD has also been shown to exhibit greater dysbiosis than UC,^87^ which may partly explain why the two conditions require different dietary strategies.

Consistent with these differences some distinct patterns emerged. Both conditions benefited from improvements in overall diet quality, as seen with MD, AID and whole food approaches that showed efficacy in CD and UC alike. Beyond this shared effect, each condition appeared to respond to targeted strategies aligned with its pathophysiology. CD responded particularly to structured exclusion protocols: the CDED, made only for CD, demonstrated high remission rates coupled with strong biomarker response. There has been interest in an analogous UC exclusion diet but has so far only been tested in a paediatric population with limited efficacy.^88^ Likewise, CD seemed to respond better than UC to a low FODMAP diet.^47^

UC appears responsive to interventions that address its underlying barrier impairment. Elevated colonic hydrogen sulfide, produced by sulfate-reducing bacteria, contributes to a disruption in the epithelial barrier integrity.^46,89^ RS diets, tested exclusively in active UC, directly target this mechanism and show feasibility evidence of clinical improvement^46^ and microbiome shifts toward greater diversity and SCFA-producing taxa.^64^ Whether analogous sulfur-mediated mechanisms operate in CD remains untested. The MD also appeared specifically promising across both active and quiescent UC.^55,56^ Together, these patterns suggest that CD may require more targeted interventions modulating specific taxa and metabolites, while UC may respond to broader strategies enhancing microbial richness and mucosal barrier integrity.^80,90^ ^86^Definitive disease-specific conclusions remain premature given the heterogeneity of the evidence, and these observations should be considered hypothesis-generating. Notably, the case studies^32,33,65,66,71^ did not reveal any unexpected findings but provide proof-of-concept evidence for sustained benefits in individual patients.

### Clinical implications: Rethinking what dietary benefit means in IBD

A recurring finding was the frequent disconnect between subjective symptomatic improvement and objective markers of inflammation. Many studies reported meaningful reductions in patient-reported symptoms and disease activity scores without corresponding changes in biomarkers such as FCP, CRP, or endoscopic assessment. As noted by Comeche et al.^25^ in their systematic review, this disconnect raises important questions about the mechanisms of action and long-term efficacy of dietary interventions. Within conventional IBD outcome frameworks, this divergence reads as partial failure. It can be argued however, that this framing deserves reconsideration.

As part of this reconsideration, it is important to mention that mucosal healing has become a primary therapeutic target in IBD management and is an appropriate endpoint for pharmacological therapies designed to directly suppress mucosal inflammation. However, applying it uncritically to dietary interventions may be conceptually misaligned. Diet is a long-duration, low-intensity, multi-mechanism intervention that likely operates through pathways such as modulation of the microbiome,^91–94^ improved barrier integrity,^95,96^ gut motility and visceral sensitivity,^78^ which do not always manifest in biomarkers or endoscopic appearance. Several explanations for the disconnect are plausible, and the timescale may simply exceed most trial durations. This is supported by RS dietary studies in UC, where significant intraluminal changes, including reduced H_2_S-producing taxa, altered sulfur-cycling gene expression, and suppressed protein fermentation, were demonstrable after eight weeks without consistently translating into endoscopic or biomarker response,^64,89^ and has been observed more broadly across immune-mediated inflammatory diseases, where symptom improvements frequently occur in the absence of corresponding biomarker changes.^97^ Marlow et al.^41^ transcriptomics showed significant changes even when biomarkers like CRP showed only non-significant trends after six weeks, suggesting that measurable biological change may be underway even when standard markers appear flat. Additionally, emerging evidence from time-restricted feeding^73^ and fasting-mimicking diet,^74^ both published outside our formal search window, point to dietary timing and caloric restriction as a promising mechanistic and clinical avenue, with the latter also demonstrating attenuation of key inflammatory lipid mediators and effector transcripts in humans with active CD. Whether these effects are driven by weight reduction itself or by weight-independent mechanisms remains an open question. Particularly given that higher BMI has separately been linked to increased autoimmune disease risk, with adipose tissue-derived immunological changes proposed as a possible contributing mechanism.^98^

For clinical practice, a diet that reduces symptoms and improves QoL is clinically valuable for patients living with a chronic relapsing condition that affects daily functioning and psychological well-being. The AGA^12^ recommends that all patients with IBD follow a Mediterranean-style diet rich in fresh fruits, vegetables, monounsaturated fats, complex carbohydrates, and lean proteins, and low in processed foods, added sugar, and salt, specifically for overall health and general well-being. The same guidelines acknowledge that no diet has consistently been found to decrease flare rates in adults with IBD. The coexistence of these two statements means that: dietary recommendation can be clinically warranted even where flare prevention is not yet reliably demonstrated. Dietary approach can also be individualised based on disease phenotype, food preferences, and practical feasibility. Considering that patient adherence is shaped by acceptability and sustainability, it may be as important as theoretical mechanistic superiority.

An important caveat accompanies this pragmatic optimism. Restrictive dietary interventions such as the SCD, AIP, 4-Sure diet (a type of RS diet) and CDED may offer clinical benefit for some patients but carry risk of nutritional deficiencies and are not appropriate for widespread recommendation without professional supervision. Professional dietary guidance by a dietitian is necessary, a concern reflected in current guidelines.^11,12^

For research design, the symptom-biomarker disconnect points to a fundamental methodological problem: the field may be measuring the wrong things, with the wrong time span, in insufficiently defined populations. Importantly, these methodological shortcomings are not new. Lewis et al.^99^ identified many of the same problems nearly a decade ago: including underpowered studies and inadequate comparator design, yet the field has been slow to act on them. This problem also seems to extend beyond IBD as a recent review of dietary meta-analyses across immune-mediated inflammatory diseases found that trials frequently grouped diets with meaningfully different compositions under the same name, lacked standardised tools to characterise the inflammatory potential of interventions, and rarely assessed inflammatory biomarkers even when symptom improvements were observed, concluding that a validated index objectively estimating the impact of a well described diet on specifically determined clinical parameters is needed to enable meaningful comparison between trials.^97^ A consensus-derived core outcome set for dietary IBD trials is needed, one that distinguishes between induction and maintenance goals and specifies when endoscopic assessment is required. Future studies should incorporate microbiome profiling, metabolomics, and intestinal permeability markers alongside clinical indices, and should treat CD and UC as distinct populations. Diet composition reporting must also become more rigorous, as the substantial variability observed even within nominally identical interventions makes replication difficult and cross-trial comparison often misleading.

What this review suggests, ultimately, is that the field does not need more trials of new diets. It needs better-designed trials of existing promising approaches in the right populations, with the right endpoints, and with sufficient follow-up to capture the timescale on which dietary change plausibly operates.

### Strengths and limitations

Dietary intervention research faces inherent methodological challenges that contextualise the limitations of any review in this area and are not unique to IBD. Blinding is rarely achievable, sample sizes are frequently small, and outcome measures are inconsistently applied across trials. Diet composition reporting is frequently inadequate, making replication difficult and cross-trial comparison often misleading.

This scoping review has several strengths. The systematic search strategy across multiple databases, inclusion of diverse study designs, and detailed charting of dietary composition with heat-map visualisation aided in identifying both consensus and divergence. Additionally, a focus on separating CD and UC provided the ability to see disease-specific patterns. A strength with the inclusion criteria is that we only included adults, which to an extent helped to limit heterogeneity as IBD manifestations and treatment responses can differ between paediatric and adult populations.

However, several limitations must be acknowledged. As a scoping review, formal quality assessment of included studies was not performed, and no meta-analysis was conducted. The heterogeneity complicated synthesis and comparison across studies. In view of the increasing numbers of articles about diet in IBD we chose year 2000 as an appropriate cut-off. This could be questioned; an earlier cut-off would yield some more articles. On the other hand, these articles were based on less knowledge about diet than we have nowadays. We estimated that the extra information we could gain would not justify an extended search.

Furthermore, the review focused on clinical outcomes rather than mechanistic studies. While this clinical focus provides practical relevance, it limits understanding of how and why certain dietary components may benefit IBD patients. Finally, despite efforts to include late adolescents alongside adults, most studies with mixed age ranges had average participant ages below 18 and could not be included.

## Conclusion

This review was conducted to inform the design of forthcoming dietary interventions, and that purpose shapes how its findings should be read. The evidence does not yet point to a single diet to implement, but it does point clearly toward the MD and shared whole-food principles as a well-supported, safe, and guideline-endorsed foundation from which to build. The next step is not to wait for certainty, but to design research rigorous enough to generate it with standardised outcomes, disease-specific populations, mechanistic sub-studies, and the implementation support that adherence requires. Patients with IBD are already modifying their diets, often without guidance. The field’s responsibility is to catch up.

## Supplemental material

Supplemental material - Multiple paths to health: A scoping review of dietary interventions for adults with Crohn’s disease and ulcerative colitis Supplemental material for Multiple paths to health: A scoping review of dietary interventions for adults with Crohn’s disease and ulcerative colitis by Josephine Ingridsdotter, Vedrana Vejzovic, Eva Elmerstig, Klas Sjöberg in Therapeutic Advances in Gastroenterology.

Supplemental material - Multiple paths to health: A scoping review of dietary interventions for adults with Crohn’s disease and ulcerative colitis Supplemental material for Multiple paths to health: A scoping review of dietary interventions for adults with Crohn’s disease and ulcerative colitis by Josephine Ingridsdotter, Vedrana Vejzovic, Eva Elmerstig, Klas Sjöberg in Therapeutic Advances in Gastroenterology.

## Data Availability

No new data were generated or analysed in support of this research.*

## Appendix

### Abbreviations

AGA: American Gastroenterological Association
AID: Anti-Inflammatory Diet
AIP: Autoimmune Protocol
CD: Crohn’s Disease
CDED: Crohn’s Disease Exclusion Diet
CRP: C-Reactive Protein
ECCO: European Crohn’s and Colitis Organisation
EEN: Exclusive Enteral Nutrition
ESR: Erythrocyte Sedimentation Rate
FCP: Faecal Calprotectin
FODMAP: Fermentable Oligo-, Di-, Monosaccharides And Polyols
HBI: Harvey-Bradshaw Index
IBD: Inflammatory Bowel Disease
IBDQ: Inflammatory Bowel Disease Questionnaire
IBS: Irritable Bowel Syndrome
IgG: Immunoglobulin G
IgG4: Immunoglobulin G4
MD: Mediterranean Diet
MRI: Magnetic Resonance Imaging
PBD: Plant-Based Diet
PEN: Partial Enteral Nutrition
PRISMA-ScR: Preferred Reporting Items for Systematic reviews and Meta- Analyses extension for Scoping Reviews
QoL: Quality of Life
RS: Reduced sulfur
SCD: Specific Carbohydrate Diet
SIBDQ: Short Inflammatory Bowel Disease Questionnaire
TIDieR: Better reporting of interventions: template for intervention description and replication
UC: Ulcerative Colitis
