# Supplementary material for: Multiple paths to health: A scoping review of dietary interventions for adults with Crohn’s disease and ulcerative colitis
Source: Ther Adv Gastroenterol. 2026 Aug 13;19:17562848261479366. doi: 10.1177/17562848261479366 (PMC13473801; doi:10.1177/17562848261479366)
Supplement: Supplementary file 2 [file sj-pdf-2-tag-10.1177_17562848261479366.pdf]

CINAHL

Original search: 251 records (2000–2025).

Updated search: 32 records (January–December 2025).

```
((MH "Diet+" OR MH "Diet Therapy" OR TI Diet OR TI Dietary OR TI "Diet
Therapy" OR AB Diet OR AB Dietary OR AB "Diet Therapy") AND (MH "Remission,
Induction" OR TI "disease activity" OR TI remission OR TI symptoms OR AB
"disease activity" OR AB remission OR AB symptoms) AND (MH "Inflammatory
Bowel Diseases+" OR TI "inflammatory bowel disease" OR TI "Inflammatory
Bowel Diseases" OR TI IBD OR TI "ulcerative colitis" OR TI "crohn* disease"
OR AB "inflammatory bowel disease" OR AB "Inflammatory Bowel Diseases" OR
AB IBD OR AB "ulcerative colitis" OR AB "crohn* disease")) AND (MH
"Adolescence+" OR MH "Young Adult+" OR MH "Adult+" OR TI teenager OR TI
teenagers OR TI teens OR TI youth OR TI "young person" OR TI "young people"
OR TI adolescence OR TI adolescent OR TI "young adult" OR TI "young adults"
OR TI adult OR TI adults OR TI "grown-up" OR TI "mature person" OR TI
"mature people" OR AB teenager OR AB teenagers OR AB teens OR AB youth OR
AB "young person" OR AB "young people" OR AB adolescence OR AB adolescent
OR AB "young adult" OR AB "young adults" OR AB adult OR AB adults OR AB
"grown-up" OR AB "mature person" OR AB "mature people"))
```

SCOPUS

Original search: 1644 records (2000–2025).

Updated search: 286 records (January–December 2025).

```
((TITLE-ABS-KEY(Diet) OR TITLE-ABS-KEY("Diet Therapy") OR TITLE-ABS-
KEY(Dietary))  AND TITLE-ABS-KEY("disease activity") OR TITLE-ABS-
KEY(remission) OR TITLE-ABS-KEY(symptoms) AND (TITLE-ABS-KEY("inflammatory
bowel disease") OR TITLE-ABS-KEY("Inflammatory Bowel Diseases") OR TITLE-
ABS-KEY(IBD) OR TITLE-ABS-KEY("ulcerative colitis") OR TITLE-ABS-
KEY("crohn* disease"))) AND (TITLE-ABS-KEY(teenager) OR TITLE-ABS-
KEY(teenagers) OR TITLE-ABS-KEY(teens) OR TITLE-ABS-KEY(youth) OR TITLE-
ABS-KEY("young person") OR TITLE-ABS-KEY("young people") OR TITLE-ABS-
KEY(adolescence) OR TITLE-ABS-KEY(adolescent) OR TITLE-ABS-KEY("young
adult") OR TITLE-ABS-KEY("young adults") OR TITLE-ABS-KEY(adult) OR TITLE-
ABS-KEY(adults) OR TITLE-ABS-KEY("grown-up") OR TITLE-ABS-KEY("mature
person") OR TITLE-ABS-KEY("mature people"))
```

PUBMED

Original search: 707 records (2000–2025).

Updated search: 118 records (January–December 2025).

```
(("Diet"[MeSH Terms] OR "Diet Therapy"[MeSH Terms] OR
"Diet"[Title/Abstract] OR "Dietary"[Title/Abstract] OR "Diet
Therapy"[Title/Abstract]) AND ("Remission Induction"[MeSH Terms] OR
"disease activity"[Title/Abstract] OR "remission"[Title/Abstract] OR
"symptoms"[Title/Abstract]) AND ("Inflammatory Bowel Diseases"[MeSH Terms]
OR "inflammatory bowel disease"[Title/Abstract] OR "Inflammatory Bowel
Diseases"[Title/Abstract] OR "IBD"[Title/Abstract] OR "ulcerative
colitis"[Title/Abstract] OR "crohn* disease"[Title/Abstract]) AND
("Adolescent"[MeSH Terms] OR "Young Adult"[MeSH Terms] OR "Adult"[MeSH
Terms] OR "teenager"[Title/Abstract] OR "teenagers"[Title/Abstract] OR
"teens"[Title/Abstract] OR "youth"[Title/Abstract] OR "young
person"[Title/Abstract] OR "young people"[Title/Abstract] OR
"adolescence"[Title/Abstract] OR "Adolescent"[Title/Abstract] OR "Young
Adult"[Title/Abstract] OR "young adults"[Title/Abstract] OR
"Adult"[Title/Abstract] OR "adults"[Title/Abstract] OR "grown-
up"[Title/Abstract] OR "mature person"[Title/Abstract] OR "mature
people"[Title/Abstract]))

AND (2000:2025[pdat])
```

Web of science

Original search: 212 records (2000–2025).

Updated search: 48 records (January–December 2025).

```
(TI=(Diet OR "Diet therapy" OR Dietary) OR AB=(Diet OR "Diet therapy" OR
Dietary)) AND (TI=("remission induction" OR "disease activity" OR remission
OR symptoms) OR AB=("remission induction" OR "disease activity" OR
remission OR symptoms)) AND (TI=("inflammatory bowel disease" OR
"Inflammatory Bowel Diseases" OR IBD OR "ulcerative colitis" OR "crohn*
disease") OR AB=("inflammatory bowel disease" OR "Inflammatory Bowel
Diseases" OR IBD OR "ulcerative colitis" OR "crohn* disease")) AND
(TI=(Adolescent OR "Young Adult" OR Adult OR teenager OR teens OR youth OR
"young person" OR "young people" OR adolescence OR "grown-up" OR "mature
person" OR "mature people") OR AB=(Adolescent OR "Young Adult" OR Adult OR
teenager OR teens OR youth OR "young person" OR "young people" OR
adolescence OR "grown-up" OR "mature person" OR "mature people"))
```
